# Comprehensive comparison of molecular portraits between cell lines and tumors in breast cancer

**DOI:** 10.1186/s12864-016-2911-z

**Published:** 2016-08-22

**Authors:** Guanglong Jiang, Shijun Zhang, Aida Yazdanparast, Meng Li, Aniruddha Vikram Pawar, Yunlong Liu, Sai Mounika Inavolu, Lijun Cheng

**Affiliations:** 1 Center for Computational Biology and Bioinformatics, School of Medicine, Indiana University, Indianapolis, IN 46202 USA; 2 Department of Medical and Molecular Genetics, School of Medicine, Indiana University, Indianapolis, IN 46202 USA

**Keywords:** Heterogeneous, Breast cancer, DNA mutation, mRNA expression, Copy number alteration, Reverse-phase protein array, Molecular portraits, Cell lines

## Abstract

**Background:**

Proper cell models for breast cancer primary tumors have long been the focal point in the cancer’s research. The genomic comparison between cell lines and tumors can investigate the similarity and dissimilarity and help to select right cell model to mimic tumor tissues to properly evaluate the drug reaction in vitro. In this paper, a comprehensive comparison in copy number variation (CNV), mutation, mRNA expression and protein expression between 68 breast cancer cell lines and 1375 primary breast tumors is conducted and presented.

**Results:**

Using whole genome expression arrays, strong correlations were observed between cells and tumors. PAM50 gene expression differentiated them into four major breast cancer subtypes: Luminal A and B, HER2amp, and Basal-like in both cells and tumors partially. Genomic CNVs patterns were observed between tumors and cells across chromosomes in general. High C > T and C > G trans-version rates were observed in both cells and tumors, while the cells had slightly higher somatic mutation rates than tumors. Clustering analysis on protein expression data can reasonably recover the breast cancer subtypes in cell lines and tumors. Although the drug-targeted proteins ER/PR and interesting mTOR/GSK3/TS2/PDK1/ER_P118 cluster had shown the consistent patterns between cells and tumor, low protein-based correlations were observed between cells and tumors. The expression consistency of mRNA verse protein between cell line and tumors reaches 0.7076. These important drug targets in breast cancer, ESR1, PGR, HER2, EGFR and AR have a high similarity in mRNA and protein variation in both tumors and cell lines. GATA3 and RP56KB1 are two promising drug targets for breast cancer. A total score developed from the four correlations among four molecular profiles suggests that cell lines, BT483, T47D and MDAMB453 have the highest similarity with tumors.

**Conclusions:**

The integrated data from across these multiple platforms demonstrates the existence of the similarity and dissimilarity of molecular features between breast cancer tumors and cell lines. The cell lines only mirror some but not all of the molecular properties of primary tumors. The study results add more evidence in selecting cell line models for breast cancer research.

**Electronic supplementary material:**

The online version of this article (doi:10.1186/s12864-016-2911-z) contains supplementary material, which is available to authorized users.

## Background

According to a recent World Health Organization report, breast cancer is the second most common type of cancer. Each year there are about 2300 new cases of breast cancer in men and 230,000 new cases in women in the U.S. [1]. While age and gender are two primary demographic risk factors in breast cancer, about 5–10 % of breast cancer risk is attributed to hereditary gene mutations in BRCA1, BRCA2 and TP53 [2]. Breast cancer is a complex disease. Its heterogeneous nature has been classified by its molecular characteristics. The protein expression status of estrogen receptor alpha (ER), progesterone receptor (PR), human epidermal growth factor receptor-2 (HER2) decide the group of breast cancers. It can be subtyped as Luminal A (ER+/PR+, HER2+), Luminal B (ER+/PR+, HER2-), HER2amp (HER2 positive) and Basal-like/triple negative (ER-,PR-, HER2-) [3, 4]. The Basal-like patients are correlated with biologically aggressive disease and often have a poor prognosis [3]. In Luminal A and Luminal B subtypes, ER was identified as the therapeutic target, and its targeted hormone therapies (such as tamoxifen and letrazole) have been well established. In HER2 amplification group, trasuszumab is the candidate drug. However, basal-like triple negative tumors still do not have recognizable therapies. The target identification and its subtype classification is an important aspect for therapy development in breast cancer [5, 6].

Cell lines, originated from human tumors, have historically acted as the primary experimental model to investigate the cancer biology and molecular pharmacology. Parallel massive drug screening on these cancer cells characterize the diverse cancer cell reactions to drugs by genomic features. As a salient example, the Cancer Cell Line Encyclopedia (CCLE) project conducts a detailed genetic characterization of a large panel of 997 human cancer cell lines in DNA copy number, mRNA expression and mutation [7]. Together with the drug screening data, CCLE becomes a powerful resource for the drug and target discovery researches.

Breast cancer is heterogeneous in nature. Cell lines study is only an interpretation from a context of artifacts introduced by selection and establishment in vitro, and there exists large differences between cancer cell lines and tissue samples especially in its molecular genome [8, 9]. Selecting the right cells model to mimic tumor tissues helps to evaluate proper drug reactions in tumors in vitro [10, 11]. Gene-expression profiling has become an important tool to characterize both the similarity and dissimilarity between cell lines and tumors. A recent work by Ross DT [12] demonstrated the distinctive gene expression signature in breast cancer tissue: basal, luminal epithelial cell signature, as well as mesenchymal/stromal. Lacroix M [13] valuated some widely used breast cancer cell lines as breast tumor models by a comparative genetic expression features. Besides gene expression, CNV has gradually been recognized as important due to features in predicting cancer progression and recurrence. Jessica Kao et al. [14] compared the gene expression profiles and CNVs of breast cancer cells and tumor tissues to define relevant cell line models. Both Fridlyand et al. [10] and Richard M. et al. [15] conducted similar analyses, in which the similarity was further investigated within the breast cancer subtypes. Nevertheless, these researches provide important information for understanding a molecular mechanism from only one aspect of the breast cancer genome, such as mRNA or DNA or protein, but not both. No one has yet attempted to investigate the correlation between cell lines and tumor tissues from all CNV, mutation, gene expression and protein expression between and within breast cancer subtypes systematically.

The Cancer Genome Atlas (TCGA) [15] aims to discover major cancer-causing genomic alterations. It publicly provides 1098 breast tumor samples with mRNA expression profiling, DNA exome parallel sequencing, CNV, and protein expression. Because of this valuable data, a number of important breast cancer genes and pathways were detected systematically during the past 3 years [16–18]. However, systematic comparisons between TCGA breast tumor samples and breast cell line data, such as Cancer Cell Line Encyclopedia (CCLE), have not yet been conducted. The primary innovation of this comparison is that, for the first time, four layers of genomic data: CNV, mutation, mRNA expression and protein expression, were investigated to seek the similarity or dissimilarity between breast cancer cells and tumors. Secondly, because of better sensitivity and broader dynamic range of sequencing technology comparing to the array platforms, genomic data was better captured in TCGA and CCLE by the platform data comparison. In this paper, a comprehensive comparison in CNV, mutation, mRNA expression and protein expression between CCLE breast cancer cell lines and TCGA primary breast tumors is presented separately. At the end, a total score that integrates four genomic features will be defined to investigate the overall similarity between breast cancer cell lines and its tumor tissues.

## Results

**Fig. 1:**
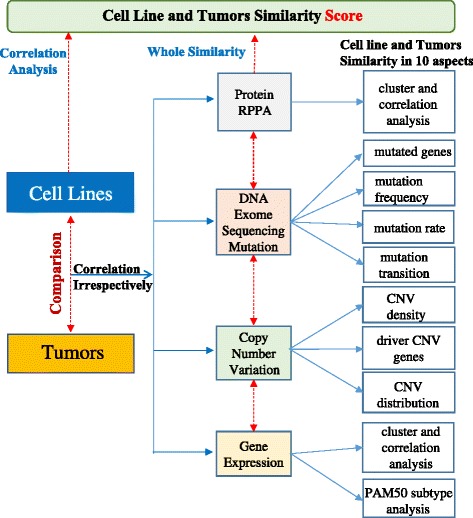
The whole analysis process between cell lines and tumors in breast cancer using 4 genomic profiles. Sixty eight cell lines and 1375 tumors are compared in gene expression, copy number variation (CNV), mutation and protein across 10 aspects. A score that integrated four genomic features was used to evaluate the overall similarity of tumors and cell lines

Sixty-eight breast cancer cell lines were extracted from CCLE [7] and literature [19]. One thousand seven hundred five breast cancer tumor samples were obtained from TCGA and Gene Expression Omnibus (GEO). All of the datasets are listed in Table 1. Different subsets of samples were assayed on four different level platforms, including Affymetrix HU133 and Agilent G4502A_07_3 for mRNA expression microarrays irrespectively, Affymetrix 6.0 single nucleotide polymorphism (SNP) arrays for copy number variation, whole-exome sequencing in TCGA and hybrid capture sequencing 1651 genes in CCLE for mutation analysis. Reverse-phase protein lysate microarrays (RPPAs) are used to test basal phosphorylation and protein abundance in TCGA tumors and cell lines. Please note that not all samples were characterized on each platform. Different subsets of tumors and cell lines were analyzed in each platform (Additional file 1: Tables S1 and S2). Each one of the four platform data analyses focused on the overlapping genes between tumors and cell lines, and the overall similarity analysis by using all four platforms was conducted afterward. Figure 1 describes the overall analysis process between cell lines and tumors in breast cancer.

**Table 1:** Four molecular profiles datasets for tumor and cell lines comparison in breast cancer

Data types	Sources	Platforms	Samples size
Copy number variation	TCGA;CCLE	Affymatrix SNP 6.0	1033; 59
Mutation (Exome Sequencing)	TCGA;CCLE	Illumina GAIIx	967; 51
Gene expression	TCGA; GEO; CCLE	AgilentG4502A_07_3 (TCGA); Affymatrix HU133 Plus 2.0 (GEO; CCLE)	530; 279; 58
Protein	TCGA; CCLE	RPPA	197; 38

### Gene expression profiles comparison between breast cancer cell lines and tumors

**Fig. 2:**
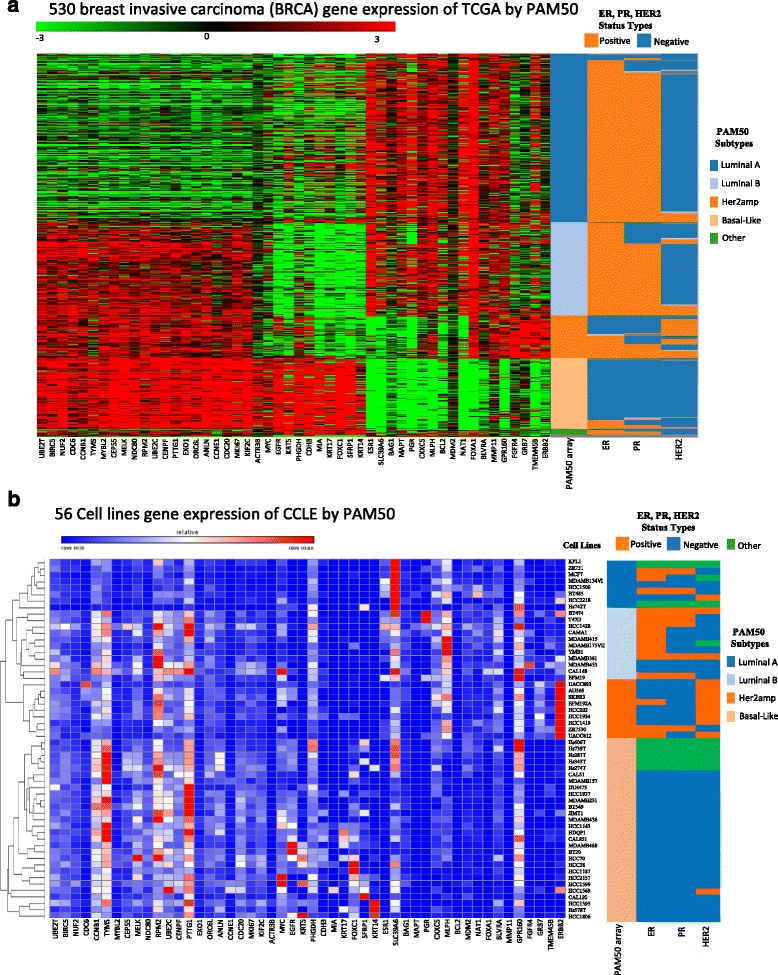
Gene expression PAM50-breast cancer subtype classifications of cell lines and primary tumors for ER, PR, Her2 status. **a** The PAM50 subtype classification of 530 invasive breast cancer samples in TCGA, which uses AgilentG4502A_07_3 Array platform. **b** The PAM50 subtype classification of 56 breast cancer cell lines, which uses Affymetrix Human Genome U133 Plus 2.0 Array platform

PAM50 (Prediction Analysis for Microarrays) [20] is one of the most common genetic tests for breast cancer subtyping. The PAM50 was designed as a RT-qPCR 50-gene expression signature. It has been acknowledged as a prognostic gene signature assay by an authoritative organization, National Comprehensive Cancer Network (NCCN) (http://www.nccn.org/), in year 2015. Due to this, many breast tumor and cell line samples lacked of ER, PR, and HER2 status for breast cancer treatment classifications. As for the missing information of HER2 status, it has 182 in 1096 TCGA tumors and 15 in 68 CCLE cell lines. These samples are classified as subtypes of Luminal A, Luminal B, HER2amp, and Basal-like using the PAM50 signature. On the other hand, the RT-qPCR and mRNA-based PAM50 ER/PR/HER2 classification results are compared. Figure 2 displays the PAM50 gene expression signature predicted subtypes of cell lines and tumors in breast cancer, and the observed ER, PR, HER2 status. Eight hundred seventy five TCGA samples have information of ER/PR/HER2 status in 1096 tumors, while 53 cell lines in 68 CCLE samples have those. Figure 2a shows the PAM50 subtypes of 530 invasive breast cancer patients in TCGA using AgilentG4502A_07_3 array platform. Comparing to the standard ER, PR, and HER2 status for classification of breast carcinoma by using immunohistochemistry staining (Table 2), 341 tumors with PAM50 classification are in concordance with the standard classification in 514 tumors, where the normal-like patients (other) are excluded. The concordance rate is 66.3 %. Figure 2b shows the breast cancer subtype classification of 56 breast cancer cell lines in CCLE using 50 genes PAM analysis. Gene expression profile in CCLE was conducted in Affymetrix Hu133 Plus2.0 Array platform. Thirty-four cell lines with known classification are in concordance with PAM50 classification, and the concordance rate is 60.71 % (34/56). Some cell lines without ER/PR/HER2 status, such as KPL1, ZR751, HS742T, HS60T, HS281T, HS343T, HS274, received ER/PR/HER2 imputation from the PAM50 prediction. In the follow-up data analysis, we kept the known classification and imputed PAM50 for both cell lines and tumor samples. Additional file 1: Tables S1 and S2 list the classification results for cell lines and tumors based on PAM50 gene expression. Interestingly, we observed that the gene expression pattern of PAM50 between cell lines and tumors are similarity, but some genes in cell lines are not as highly expressed as in tumors, such as gene FOXA1 and ESR1.

**Table 2:** Molecular classification of breast carcinoma

Classification	Immunoprofile	Other characteristics
Luminal A	ER+/PR+/HER2-; ER+/PR-/HER2-; ER-/PR+/HER2-,	Low tumor grade, Low expression of proliferation marker Ki67
Luminal B	ER+/PR+/HER2+; ER+/PR-/HER2+; ER-/PR+/HER2+	High tumor grade, High expression of proliferation marker Ki67
HER2-enrichment	ER-/PR-/HER2+; ER-/PR-/HER2+; ER-/PR-/HER2+	High tumor grade, High expression of proliferation marker Ki67
Basal-Like	ER-/PR-/HER2-	High tumor grade, High expression of proliferation marker Ki67

**Fig. 3:**
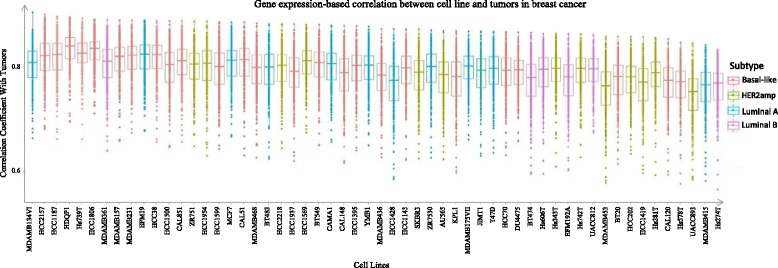
Whole genome expression correlation analysis between 56 breast cancer cell lines and 279 primary tumors. The *x*-axis indicates 56 cell lines, and *y*-axis is the correlation coefficient between tumors and cells. The cell line subtypes are denoted in different colors

In order to compare the similarity of the whole genome expression profiles between primary breast cancer tumors and breast cancer cells (i.e. CCLE samples), the breast cancer tumors in Gene Expression Omnibus (GEO) GSE41998 (279 tumors) were selected because they shared the same Affymetrics gene expression platform (Additional file 1: Table S3). Figure 3 shows the correlation distributions of whole genome expression between breast cancer cell lines and primary tumors. The 56 box plots of the correlations illustrate the similarity between 56 cell lines and 279 tumors. The correlation coefficient is around 0.6–0.8 between cell lines and tumors. These results show that cell lines keep a high similarity to tumors in whole gene expression profile in breast cancer even though in different subtypes.

### Copy number variations comparison between CCLE breast cancer cell lines and TCGA breast cancer tumors

**Fig. 4:**
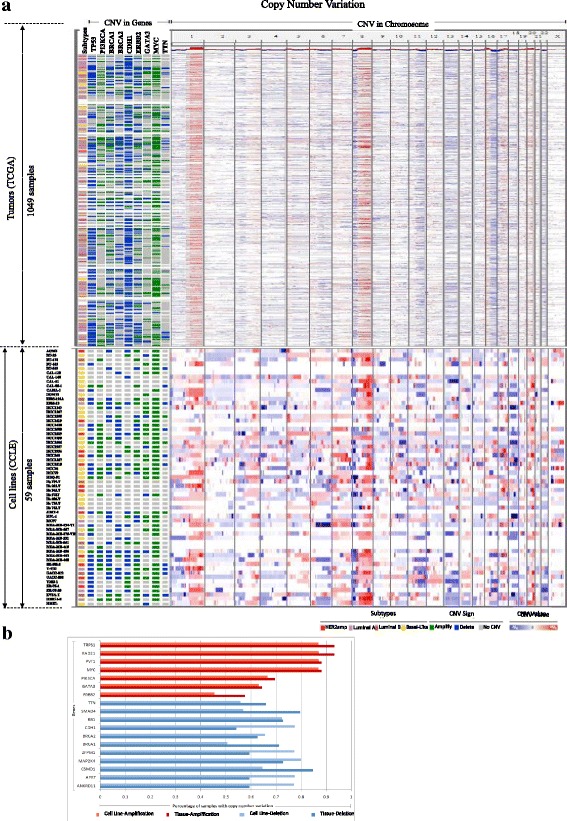
The CNV comparison between 1049 TCGA breast cancer samples and 59 CCLE breast cancer cell lines. **a** DNA copy number profiles across the whole chromosomes (right) and copy number amplification and deletion in highly mutated breast cancer driver genes. The red color indicates amplification, while the blue color indicates deletion. The top panel is TCGA, and the bottom panel is CCLE. **b** is the CNV frequency comparison for highly mutated genes between TCGA breast cancer samples and CCLE breast cancer cell lines

CNVs are compared between CCLE breast cancer cell lines and TCGA breast cancer primary tumors in various breast cancer subtypes. Figure 4 displays copy number distribution for both tumors and cell lines across 24 chromosomes. In Fig. 4a, chromosome 1 and 8 have the highest copy number amplification frequencies while chromosomes 13 and 16 have the most copy number deletion regions in both cell lines and tumor tissues. Figure 4b displays the significant genomic alterations in breast cancer tumors and cell lines. MYC, PVT1, RAD21 and TRPS1 are top four copy number amplified genes, while MAP2K4, ANKRD11, APRT, CSMD1 and ZFPM1 are top five genes with copy number deletions. Some important cancer genes, such as PIK3CA, BRCA1, BRCA2, and ERBB2, show a mixture of amplifications and deletions.

**Fig. 5:**
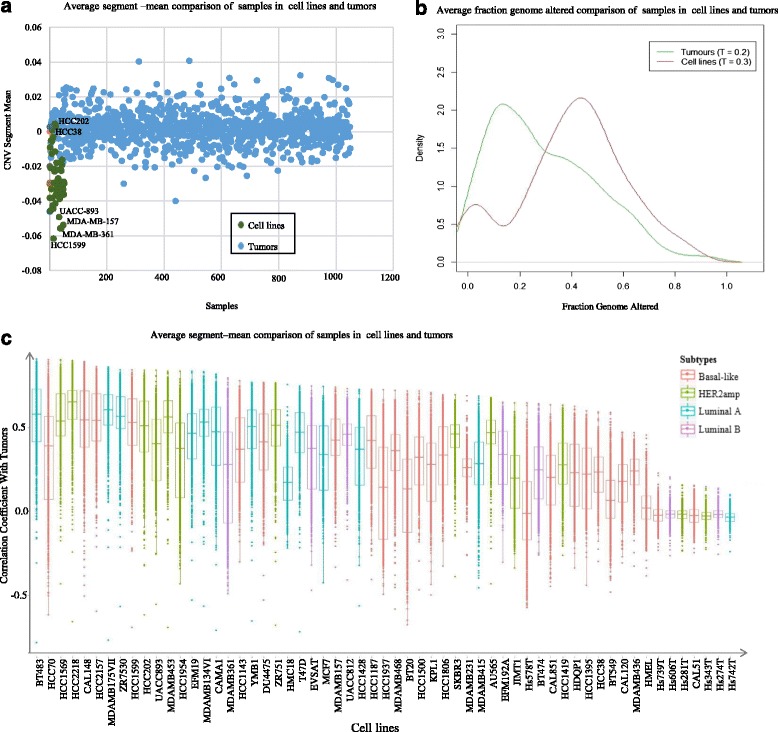
CNV similarity analysis between cell line and tumors in breast cancer. **a** is the average segmentation length comparison between cell lines and tumors samples, here the segmentation length is measured according to *log*_2_(*CN*/2)). Positive number means amplification, and negative number means deletion; **b** shows the density distribution of copy number variation in breast tumors and cell lines; **c** shows CNV-based similarity between cell line and tumors in breast cancer according to Pearson correlation coefficient using the top 10 % genes (2094 genes)

The CNVs between cell lines and tumor samples of breast cancer are compared in sample segmentation mean and density calculation of copy number Fraction Genome Altered (FGA). Its calculation is presented in the method section. Figure 5a demonstrates that cell lines have more copy number deletions than tumors. In particular, HCC1599, MDA-MB-361, MDA-MB-157, and UACC893 are the top 4 CNV deletions cell lines. In Fig. 5b, it is evident that the frequency of copy number alteration are significantly higher in cell lines than in tumors. The mean cell line FGA is wider than that of tumor FGA. In order to evaluate the similarity between tumors and cell lines, the Pearson correlations for the top 10 % CNV in 2094 genes are calculated between 59 cell lines and 1049 tumors. Fig. 5c shows the CNV-based correlation coefficient distribution between cell lines and tumors in different breast cancer subtypes. We observe that cell lines HCC2218, MDA-MB-175-VII, ZR-75-30, BT-483, HCC1569 and MDA-MB-453 are more similar to tumors in CNV than the other cancer cells. Their correlation coefficients are larger than 0.55 (*p* < 10^−18^). On the other hand, HMEL, Hs 578 T, Hs 274.T, Hs 606.T, Hs 281.T, Hs 739.T, CAL-51, Hs 343.T and Hs 742.T had negative correlation coefficients with tumors samples (*p* < 10^−2^).

### Mutation analysis in cell lines and tumors

CCLE sequenced only 1347 cancer genes in breast cancer, while TCGA has whole exome sequencing. Our comparative analysis is only based on those 1347 overlapping genes and their somatic mutations. In CCLE, in order to remove background germline mutation, mutations reported in the 1000 Genome Project and dbSNP were filtered out using ANNOVAR tool, including the gene-based single nucleotide variants (SNVs) and insertions/deletions [21].

**Fig. 6:**
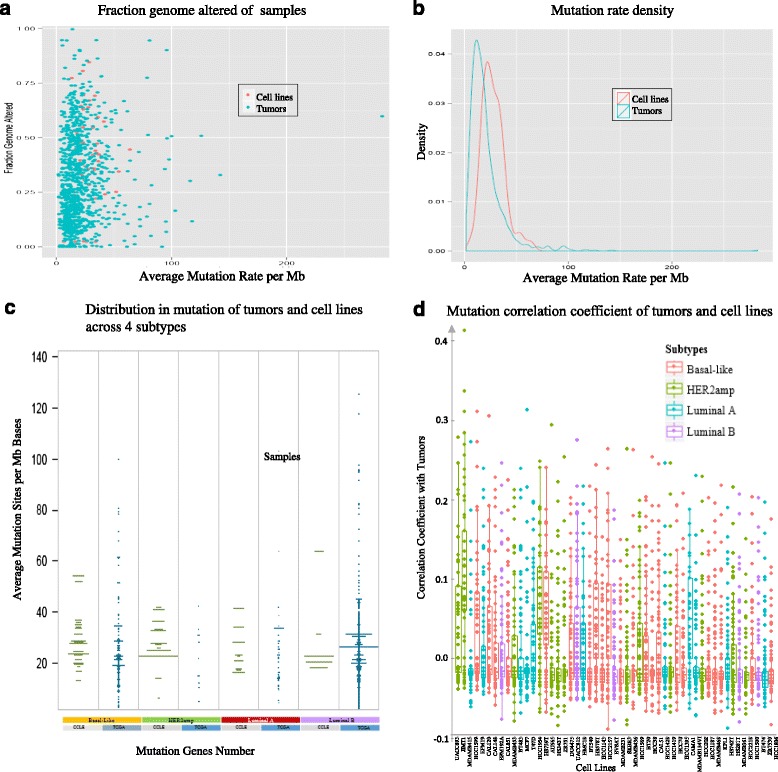
DNA sequencing-based mutation comparison between CCLE 51 cell lines and TCGA 977 tumors. **a** The scatter plot of fraction genome altered and mutation per million bases for TCGA samples and CCLE samples; **b** The mutation densities in breast tumors and cell lines; **c** Mutation-based subtypes similarity of cell line and tumors in breast cancer; **d** Mutation correlation coefficient distributions between cell lines and tumors

Figure 6 shows the comparisons of somatic mutations between cell lines and tumors across four aspects: somatic mutation frequency, somatic mutation density, average mutation sites distribution per million bases (Mb) in four subtypes, as well as mutation correlation variation between cell lines and tumors. Figure 6a illustrates the mutation frequency per Mb in TCGA and CCLE vs CNV fraction genome alteration. A subset of cell lines with hyper-mutated genes is revealed, such as MDAMB361, BT474, MDAMB453 and HCC1569. These cells of breast cancer show moderately higher mutation frequency than the tumors. Figure 6b shows the somatic mutation density. The median somatic mutational frequency for tumors in TCGA is around 13, while cell lines in CCLE is around 25. Figure 6c shows the somatic mutation distribution among four subtypes of breast cancer in TCGA and CCLE, where *y*-axis is the mutation rate per million bases and *x*-axis is mutation gene numbers. The wider the line is, the more the gene mutation number of samples is. It suggests that the gene mutation number in Luminal B subtype from TCGA is the largest. At the same time, its mutation rate is also higher than the other subtypes. Tumor and cell lines with Luminal A subtype have the lowest mutation numbers and mutation rate. Her2 subtype group in cell lines has a larger mutation number than the other subtypes. Figure 6d shows the 1347 somatic mutation genes-based correlation coefficient distributions between cell lines and tumors in different breast cancer subtypes. These genes were firstly denoted as 0 or 1 to illustrate non-mutation or mutation. The correlation is distributed in the range of [-0.1, 0.43]; Additional file 2: Table S7 shows the detail correlation coefficient between cell lines and tumors in four levels for gene expression, mutation, copy number variation and protein irrespectively. The top four cell lines that have the highest mutational correlation with tumors are: UACC893, JIMT1, EFM19 and HCC1954. The highest consistency coefficient is 0.4258.

**Fig. 7:**
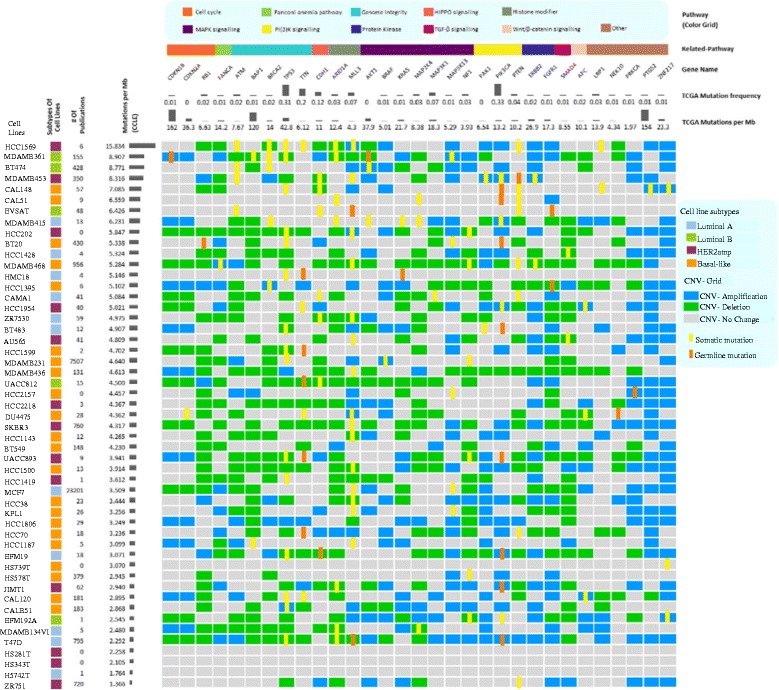
The landscape of functional driver mutations in cell lines of breast cancer. Upper rows show the gene mutation frequency and mutation rate per million bases (Mb) in 967 tumors. Left column shows the popularity of breast cancer cell lines denoted by the publication citation number in Pubmed and mutated rate per Mb in cell lines. Point mutations (germline mutation and somatic mutation) and copy number variation (CNV amplification is segment-mean > 0.3, CNV deletion is segment-mean < -0.3) are shaped into the horizontal bar and vertical bar with different color, respectively

Thirty-one genes, reported in recent TCGA nature and science papers [16–18, 22–28], were selected as important driver mutation genes in the breast cancer. These genes were further investigated across 51 breast cancer cell lines. Figure 7 shows a landscape of these functional driver mutations in these cell lines of breast cancer. According to the mutation per megabyte base calculation, HCC1569, MDAMB361, and BT474 are hyper-mutated cell lines, while HS 281 T, HS 343 T, and ZR 751 are lowly mutated cell lines. The popular cell lines MCF7 and MDAMB231 have median mutation rates. The top mutated genes in breast cancer tumors are TP53 (31 %) and PIK3CA (33 %). TP53 has copy number deletion in almost all cell lines, and has mixed somatic mutation. CNV has a dominant role in PIK3CA across 19 cell lines with mixed somatic mutations. Genome integrity pathway genes, ATM, BAP1, BRCA2, TTN and TP53, almost all have strong gene copy number amplification in cell lines mixed with somatic mutation, except for TTN. Similar data has been observed in genes MAP2K4 and MAP3K1 on MAPK signaling pathway. Genes PRKCA, PTGS2 and ZNF217 have many copy number deletions. The important drug biomarkers BRAF and ERBB2 (HER2) are relatively conservative, which do not have much somatic mutations.

**Fig. 8:**
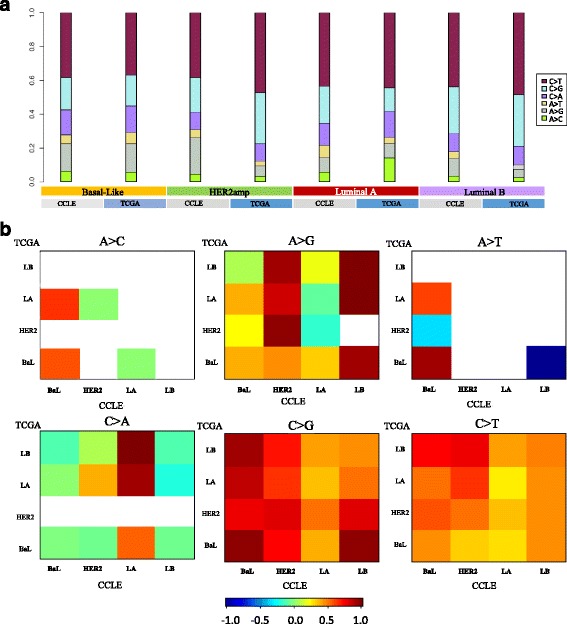
Mutation spectra and contexts across 4 subtypes of breast cancer. **a** Mutation spectrum of six transition (Ti) and transversion (Tv) categories for each subtypes of breast cancer (Luminal A = LA, Luminal B = LB, HER2amp = HER2 and Basal-Like = BaL). **b** Hierarchically clustered mutation context (defined by the proportion of A, T, C and G nucleotides within + -2 bp of variant site) for six mutation categories. Colour denotes degree of correlation: red (*r* = 1), yellow (*r* =0.5), green (*r* = 0), blue (*r* = -1)

A comparison of mutation spectra across four subtypes (Fig. 8) reveals that the mutation transition rates of cell lines and tumors are similar within different subtypes. On the other hand, it can be observed that breast cancer contains larger C > T and C > G trans-versions in subtypes HER2amp and Luminal B. HER2amp has the highest C > T trans-version rate. Luminal A has the highest A > C trans-version. Figure 8b shows the correlation of six mutation categories in tumors and cell lines. It suggests that C > T and C > G trans-version possess the highest concordance between tumors and cell lines. Basal-Like subtypes between cells and tumor tissue are consistent in A > T and C > G transition, while only A > G trans-version showed the correlation between tumors and cell lines in subtypes of Luminal B.

### Comparison analysis of proteins phosphorylation expression between cell lines and tumors in breast cancer

**Fig. 9:**
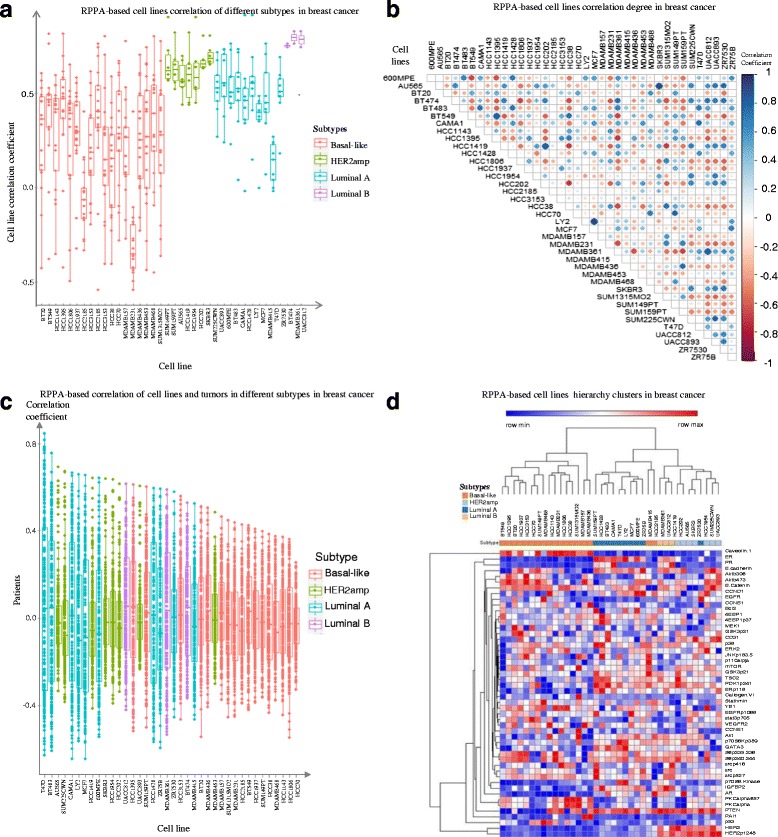
Protein-based comparison of cell lines and tumors in breast cancer according to 50 proteins in RPPA testing. **a** RPPA-based Pearson correlation distribution in different subtypes of cell lines in breast cancer. **b** RPPA-based cell lines correlation in breast cancer by Pearson correlation. The dot size expresses the correlation strong or weak, and the larger means it has a strong correlation. The color bar shows the positive or negative directions. **c** RPPA-based correlation distribution between cell lines and tumors in breast cancer by 50 phosphorylated proteins. A dot means a correlation coefficient between a cell and a tumor. The different colors represent the different breast cancer subtypes. The same subtype of tumors and cells are used to calculate their correlation coefficient. **d** RPPA-based cell lines hierarchy clusters in breast cancer. Rows are proteins while columns are cell line samples

Quantitative expression of 50 cancer-related proteins, phosphorylated-proteins by RPPA, were measured on 197 breast tumors and 38 cell lines. Pearson Correlation analysis and unsupervised hierarchical clustering analyses were conducted between cell lines and tumors (Fig. 9). The correlations in Fig. 9a suggest that all four cell line subtypes possess different correlation distributions with tumor samples. Luminal B cells have the highest correlations, while basal cells have the lowest correlations and also show the largest variations. Figure 9d illustrated hierarchy distance among cell lines. It suggests that the same subtype cell lines usually are closely clustered. Protein expressions for ER and PR have high concordance, and they are reversely correlated with Coveolin.1 in all subtypes, especially in the Basal-Like subtype. A similar variation phenomena was observed in several other groups’ of proteins in different subtypes: (EGFA, CCNB1), (4EBP1, MEK1), (mTOR, GSK3), and (GATA3, p70s6kp389, AKT). Correlations between cell lines and tumors are further illustrated in Fig. 9c. The correlation ranges from −0.61 to 0.84. Some cell lines, T47D, BT483, and AU565, are the top three cell lines that have closer correlations to tumors in protein level, while the most popular breast cancer cell line, MCF7, is somewhere in the middle. The exact correlations between cell lines and tumors are presented in Additional file 1: Table S6 based on 50 phosphor-proteins.

**Fig. 10:**
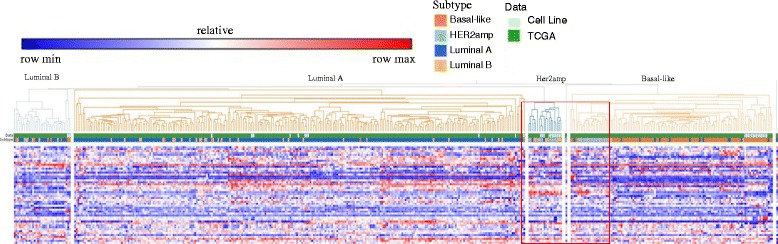
50 protein RPPA-based hierarchical clustering between 197 tumors and 38 cell lines. Rows are different proteins and columns are tumors and cell lines samples. Two color bars represent subtypes of breast cancer and data types irrespectively

Figure 10 shows the hierarchical distance between cell lines and tumors based on the 50 phosphorylated-proteins. The cell lines and tumors are assembled together by these proteins. It clearly classifies these breast cancer samples into four distinctive subtypes. Interestingly, the Basal-like cell lines MDAMB436, SUM139PT and HCC2185 are similar to protein features of Luminal A subtypes in tumors. Another discovery is that the Basal-like cell line MDAMB453 is close to Luminal B tumors. All details of the result is referred to in Additional file 2: Table S7, protein RPPA correlation coefficient between cell lines and tumors.

### Correlation analysis of gene expression verse phosphorylated protein expression between cell lines and tumors in breast cancer

**Fig. 11:**
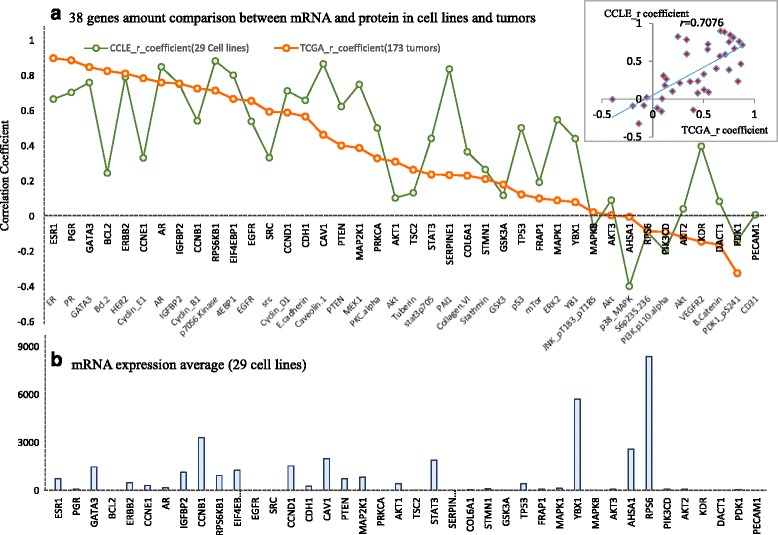
The comparison between 38 genes mRNA expression and their phosphorylated proteins expression in tumors and cell lines. **a** The correlation comparison of mRNA verse phosphorylated protein in cell lines and tumors. **b** The 38 gene expression average in 29 cell lines

The correlations of the gene mRNA versus its phosphorylated protein was calculated in cell lines and tumors irrespectively. The average correlation coefficient (Fig. 11) of 38 genes’ mRNA with their 50 phosphorylated proteins concentration ranges from −0.3 to 0.9 both in cell lines and tumors. Nearly 60% of the genes had a positive correlation between mRNA and protein. ESR1 has the highest correlation coefficient *r*−0.89 in 173 TCGA tumors, and *r* = 0.68 in 29 CCLE cell lines of breast cancer between mRNA and protein. Drug-target genes, such as PGR, HER2, EGFR and AR, all have high correlation (*r* > 0.5, *p* < 0.01) between mRNA and protein both in TCGA tumors and cell lines. Two important oncogenes, GATA3 and RP56KB1, both have high mRNA- protein correlation. The correlation for GATA3 is 0.79 in cell lines and 0.81 in tumors, while the correlation for RP56KB1 is 0.92 in cell lines and 0.78 in tumors. The small figure in Fig. 11a shows the linear correlation of the gene-protein between cell lines and tumors, which the linear correlation coefficient is 0.7076 (*p* < 0.01). This strong signal indicates the consistency of gene expression and protein expression in both cell line and tumor. The potential discrepancy could be due to the stability of mRNA, the degradation of protein, the time dependent and site dependent nature of protein phosphorylation, and etc. The interesting result in the Fig. 11b illustrates the gene expression amount are irrelevant to the correlation of mRNA-protein. As a matter of fact, the highest expressed gene RP56 has a negative correlation with mRNA-protein correlations in both cell lines and tumors.

### What kinds of cell lines are close to tumors?

**Fig. 12:**
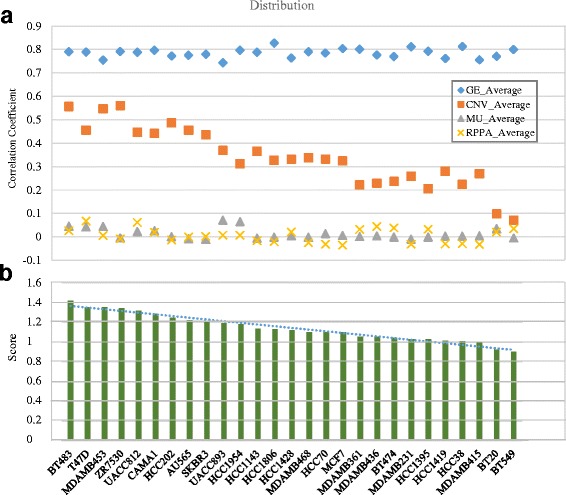
The cell lines correlation degree with tumors in 4 molecular levels to breast cancer. **a** The correlations in 4 different molecular datasets. (GE = genes expression, CNV = copy number variation, MU = DNA exome sequencing mutation, PRRA = proteins phosphorylation expression). **b** Whole score between cell lines and tumors according to 4 different molecular dataset’s correlation

Gene expression profiles and proteins phosphorylation expressions of tumors and cell lines were compared to further corroborate our observations made on the CNV and mutation data. The correlations of four different molecular profiles of all cell line and tumor pairs were calculated (Fig. 12a). These four correlations differ greatly from each other. Gene expression-based correlation had the largest correlation, CNV correlation was the next highest, mutation and protein expression correlations were low. These four correlations were combined into a total score as formula (2). Figure 12b shows the ranked cell lines by their average total correlations with the tumors. BT483, T47D, MDAMB453 are the true top 3 cell lines in breast cancer research.

## Discussion

Breast cancer is a highly complex disease. The subsets of breast tumors show diverse patterns of gene expression, CNV, mutation, and protein expression. A considerable amount of knowledge on breast carcinomas have been derived from in vivo and in vitro studies performed on breast cancer cell lines. Whether breast cancer cells are representative of the tumors remains debatable. In this study, the comparisons between cell lines and primary tumors from molecular profiles: gene expression, CNV, mutation, and protein expression, show that the cell lines are similar to some but not all of the primary tumors. Among them, gene expressions have the highest while the mutation-based correlation was the lowest.

- In gene expression-based clustering analysis, cell lines possess similar clustering as with tumors using PAM50. At the same time, cell lines show stable genomic and expression patterns, as well as high correlation, with tumors in whole gene expression profile.
- From the mutation comparison between cell lines and tumors, some common features were found: the chromosome 1 and 8 regions show high frequency copy number amplification, and chromosome 13 and 16 display high frequency deletions. Some significant cancer-related genomic alterations: MYC, PVT1, RAD21, TRPS1, CDH1, RB1, PIK3CA, MAP2K4, and ANKRD11, are identified in both breast cancer tumors and cell lines. The results were verified partially in reference [10].

In the single point mutation comparison, the six trans-version distribution modes of mutation spectrum demonstrates the similarity between tumors and cell lines in four breast cancer subtypes. High frequent C > T and C > G transitions are observed in both tumors and cell lines, while few A > T happens; Basal-like tumors and cells show the high concordance. These results were confirmed by Philip J. et al. [22]. They suggested that the underlying mutation mechanism is related to transcription-coupled nucleotide excision repair (NER). NER removes bulky DNA adducts that distort the DNA double helix and introduces a strand bias for mutation. However, little is known about the trans-version processes of mutation.

In analyzing the cancer landmark genes, gene PIK3CA and TP53 in cell lines are the top 2 mutated genes that tumors have [26]. In addition, Luminal A subtype in cell lines possess hyper mutations in three genes GATA3, PIK3CA, and MAP3KI. HER2 subtype cell lines have 72 % and 39 % mutation rates for TP53 and PIK3CA, respectively. In the recent report [26], similar results in tumors were reported, in which Luminal A is dominated with a high PIK3CA mutation frequency and Luminal B had high PIK3CA and TP53 mutation frequency. HER2 cell lines have a high PIK3CA and TP53 mutations frequency in company with HER2 amplification [26]. In addition, important drug biomarkers, such as BRAF, ERBB2 (HER2), KRAS, have very low somatic mutation. All these evidences suggest that the cancer cell lines have very similar CNVs and gene mutations patterns as tumors.

On the other hand, cell lines have more genetic aberrations than primary tumors. Amplification, deletion and mutation are more frequent in the cell lines than in the tumors. This is consistent with a similar study in ovarian cancer [8]. One potential interpretation is that cell lines may have transformed numerous passages over the period of cell culture time or get contaminated with stromal cells [10]. Another interpretation could be that the cell line is derived predominantly from early-stage tumors or pleural effusions [10].

c) In protein expression-based comparison, breast cancer subtype proteins ER, PR and HER2 have a high consistence in cell lines and in tumors. RPPA can identify breast cancer subtypes clearly and accurately not only in cell lines but also in tumors according to these protein statuses. RPPA is a sensitive and accurate technology to evaluate protein expression and activities. It helps the target identification, validation, and drug discovery [29, 30]. Some cell lines, T47D, BT483, and AU565, have much closer protein expression than the popular MCF7 cell does. On the other hand, protein expression correlation between cell lines and tumors in breast cancer vary greatly ranging from −0.1 to 0.4, it is also true in the same subtype cell lines and the variation is particularly high investigated in the basal-like subtype. The results were supported by Sorger et al. [31], who investigated the immediate-early signaling that regulates the AKT (AKT1/2/3) and ERK (MAPK1/3) pathways in different breast cancer cell types. They found that cell lines have diverse to ligand sensitivity and signaling biochemistry. In addition, they found that the basal-like cells have the largest variations in responding to growth factors while HER2amp cell lines have the least variations [31]. Basal-like breast cancer is a highly heterogeneous group without proper drug targets yet. Brian D. et al. investigated the subtypes for basal-like breast cancer and preclinical models for targeted therapy selection [5]. According to BRCA1, AR, PIK3CA and PTEN mutations, drugs are selected in cell lines to predict preclinical TNBC targeted therapies.
d) There are many complicated post-transcriptional mechanisms in turning mRNAs into proteins. According to correlation analyses between gene expression and phosphorylated protein expression in both cell lines and tumors, significant results are found that important drug targets in breast cancer, such as ESR1, PGR, HER2, EGFR and AR show high correlated mRNA and protein levels. High mRNA-protein correlation. Two oncogenes GATA3 and RP56KB1 with high consistency correlation between mRNA and protein expression become a promising potential drug targets. On the other hand, the gene expression variation at the mRNA level is not necessarily consistent with its protein level, such as genes TP53, KDR, DECAM1, which has been well documented in the literature [32, 33]. Most interestingly, the mRNA-protein correlation patterns comparing cell lines with primary tumors show a great deal of consistency among 38 investigated genes. However, the gene expression amount is irrelevant to the translation processing from mRNA to protein directly.
e) In the whole score overall comparison, cell lines and tumors show high gene expression-based correlations, but the correlations in mutation and protein expression level are low. The possible reason is that mutation data is discrete, and mutation rate is low.

According to PubMed search builder (http://www.pubmed.org) in year 2015, the number of citations for all breast cancer cell lines at CCLE is sorted (see Fig. 7). The most commonly studied cell lines are MCF-7, MDA-MB-231, MDA-MB-468 and SK-BR-3. They each have more than 600 PubMed citations. However, the correlation between these cell lines and tumors lies in the middle according to a total score of four molecular profile analyses. On the other hand, less popular cell lines, such as BT483, T47D, MDAMB453, are in the top 3 for representing breast tumors.

f) Breast cancer subtypes in tumors and cell lines. The breast cancer cell line classification provides a cell modeling system to primary tumors. Our study addresses the classification results for cell lines and tumors based on PAM50 (Additional file 1: Table S1 and S2). Although some classification results are not consistent with the known classification in cell lines and tumors, the whole subtype’s concordance reaches more than 60 %. Any cell line’s usage as a tumor’s model depends upon its subtype’s speculation. A hypothesis based on gene expression will lead to different cell selection versus another hypothesis based on mutation.

## Conclusion

In this paper, a comprehensive comparison in CNV, mutation, mRNA expression and protein expression between CCLE breast cancer cell lines and TCGA primary breast tumors is conducted and presented. The following are our primary conclusion. (1) PAM50 gene expression differentiated four major breast cancer subtypes, such as Luminal A and B, HER2amp, and Basal-like, in both cells and tumors. Using whole genome expression arrays, strong correlations are observed between cells and tumors. (2) Consistent CNV patterns are observed between tumors and cells across the chromosome. High C > T and C > G trans-version rates are observed in both cell lines and tumors, while cells have slightly higher somatic mutation rates than tumors. (3) Although the ER/PR/HER2 show the consistent patterns between cells and tumors, the other proteins in the RPPA platforms do not. Clustering analysis on protein expression data can reasonably recover the breast cancer subtypes in both cells and tumors. However, low correlations were observed between cells and tumors in phosphorylated proteins. (4) Nearly 50 % gene expressions are not consistent with their protein levels both in tumors and cell lines. The high and low of gene expression is irrelevant to the translation processing from mRNA to protein directly. Nevertheless, important drug targets in breast cancer, such as ESR1, PGR, HER2, EGFR and AR possess highly correlated in mRNA-protein expression both in tumors and cell lines. (5) A total similarity score developed from the four correlations among four molecular profiles suggests that cell lines, BT483, T47D and MDAMB453 have the highest similarity with tumors.

## Methods

### Data collection

Four levels of molecular profiles: mRNA gene expression, CNV, mutation, and protein expression, were retrieved from TCGA, CCLE and GEO (Table 1). The study cohort of breast cancer consists of 1375 patients and 68 cell lines. Tumors data and annotations were downloaded from TCGA data portal (https://gdc-portal.nci.nih.gov/) with tumor matched selections and level 3 data. DNA exome sequencing data was available from 967 tumors. mRNA expression by AgilentG4502A_07_3 platform test was collected for 530 samples, while copy number alteration was detected using Affymetrix 6.0 single nucleotide polymorphism array (SNP- array) in 1033 tumors, and protein expression by RPPA in 197 tumors was obtained. The total number of breast cancer cell lines in CCLE was 59 [7, 13]. DNA copy number data (59 cell lines), mutation data (51 cell lines), mRNA expression data (56 cell lines) and their annotations originate from CCLE websites (http://www.broadinstitute.org/ccle). According to reference [26], 38 cell lines of RPPA data was downloaded. ER, PR, and HER2 genes statuses in cell lines are found from references [5, 10, 34–36]. To compare the mRNA expression values between cell lines and tumors of breast cancer, the same platform datasets in tissue were downloaded from the GEO data set (GSE41998). It consisted of 279 tumor samples [37] with the entity histopathology information. Table 3 shows all of the cell lines samples annotation and classification information which used in this paper. Additional file 1: Tables S1–S3 lists all samples annotation of cell lines and patients in this paper.

**Table 3:** Cell lines annotation of breast carcinoma

Cell line name	Gender	Hist subtype1	Source	ER	PR	Her2	PAM50 mRNA	Our classification
AU565	F		ATCC	-	-	+	Her2amp	Her2amp
BT-20	F	ductal_carcinoma	ATCC	-	-	-	Basal-like	Basal-like
BT-474	F	ductal_carcinoma	ATCC	+	+	+	Luminal B	Luminal B
BT-483	F	ductal_carcinoma	ATCC	+	+	-	Luminal A	Luminal A
BT-549	F	ductal_carcinoma	ATCC	-	-	-	Basal-like	Basal-like
CAL-120	F		DSMZ	-	-	-	Basal-like	Basal-like
CAL-148	F	ductal_carcinoma	DSMZ	-	-	-	Luminal B	Basal-like
CAL-51	F		DSMZ	-	-	-	Basal-like	Basal-like
CAL-85-1	F		DSMZ	-	-	-	Basal-like	Basal-like
CAMA-1	F		ATCC	+	-	-	Luminal B	Luminal A
DU4475	F		ATCC	-	-	-	Basal-like	Basal-like
EFM-19	F	ductal_carcinoma	DSMZ	+	+	-	Luminal B	Luminal A
EFM-192A	F		DSMZ	+	-	+	Her2amp	Luminal B
EVSA-T	F		DSMZ	+	-	+	NON	Luminal B
HCC1143	F	ductal_carcinoma	ATCC	-	-	-	Basal-like	Basal-like
HCC1187	F	ductal_carcinoma	ATCC	-	-	-	Basal-like	Basal-like
HCC1395	F	ductal_carcinoma	ATCC	-	-	-	Basal-like	Basal-like
HCC1419	F	ductal_carcinoma	ATCC	-	-	+	Her2amp	Her2amp
HCC1428	F		ATCC	+	+	-	Luminal B	Luminal A
HCC1500	F	ductal_carcinoma	ATCC	-	-	-	Luminal A	Basal-like
HCC1569	F	metaplastic_carcinoma	ATCC	-	-	+	Basal-like	Her2amp
HCC1599	F	ductal_carcinoma	ATCC	-	-	-	Basal-like	Basal-like
HCC1806	F	ductal_carcinoma	ATCC	-	-	-	Basal-like	Basal-like
HCC1937	F	ductal_carcinoma	ATCC	-	-	-	Basal-like	Basal-like
HCC1954	F	ductal_carcinoma	ATCC	-	-	+	Her2amp	Her2amp
HCC202	F	ductal_carcinoma	ATCC	-	-	+	Her2amp	Her2amp
HCC2157	F	ductal_carcinoma	ATCC	-	-	-	Basal-like	Basal-like
HCC2218	F	ductal_carcinoma	ATCC	-	-	+	Luminal A	Her2amp
HCC38	F	ductal_carcinoma	ATCC	-	-	-	Basal-like	Basal-like
HCC70	F	ductal_carcinoma	ATCC	-	-	-	Basal-like	Basal-like
HDQ-P1	F	ductal_carcinoma	DSMZ	-	-	-	Basal-like	Basal-like
HMC-1-8	F		HSRRB				NON	Luminal A
Hs 274.T	F		ATCC				Basal-like	Luminal B
Hs 281.T	F		ATCC				Basal-like	Her2amp
Hs 343.T	F		ATCC				Basal-like	Her2amp
Hs 578 T	F	ductal_carcinoma	ATCC	-	-	-	Basal-like	Basal-like
Hs 606.T	F		ATCC				Luminal A	Luminal B
Hs 739.T	F		ATCC				Basal-like	Basal-like
Hs 742.T	F		ATCC				Luminal A	Luminal A
JIMT-1	F	ductal_carcinoma	DSMZ	-	-		Basal-like	Her2amp
KPL-1	F	ductal_carcinoma	DSMZ				Luminal A	Basal-like
MCF7	F		ATCC	+	+	-	Luminal A	Luminal A
MDA-MB-134-VI	F	ductal_carcinoma	ATCC	+	-		Luminal A	Luminal A
MDA-MB-157	F	ductal_carcinoma	ATCC	-	-	-	Basal-like	Basal-like
MDA-MB-175-VII	F	ductal_carcinoma	ATCC	+	-		Luminal B	Luminal A
MDA-MB-231	F		ATCC	-	-		Basal-like	Basal-like
MDA-MB-361	F		ATCC	+	+	+	Luminal B	Luminal B
MDA-MB-415	F		ATCC	+	-	-	Luminal B	Luminal A
MDA-MB-436	F		ATCC	-	-	-	Basal-like	Basal-like
MDA-MB-453	F		ATCC	-	-	-	Luminal B	Her2amp
MDA-MB-468	F		ATCC	-	-	-	Basal-like	Basal-like
SK-BR-3	F		ATCC	-	-	+	Her2amp	Her2amp
T-47D	F	ductal_carcinoma	ATCC	+	+	-	Luminal B	Luminal A
UACC-812	F	ductal_carcinoma	ATCC	+	-	+	Her2amp	Luminal B
UACC-893	F	ductal_carcinoma	ATCC	-	-	+	Her2amp	Her2amp
YMB-1	F		HSRRB	+	-	-	Luminal B	Luminal A
ZR-75-1	F	ductal_carcinoma	ATCC				Luminal A	Her2amp
ZR-75-30	F	ductal_carcinoma	ATCC	+	+	-	Her2amp	Luminal A
HCC2185				-	-	-	NON	Basal-like
HMEL							NON	Basal-like
HCC3153				-	-	-	NON	Basal-like
ZR75B				+	-	-	NON	Luminal A
600MPE				+	-	-	NON	Luminal A
SUM1315MO2				-	-		NON	Basal-like
SUM149PT				-	-	-	NON	Basal-like
SUM159PT				-	-	-	NON	Basal-like
SUM225CWN				-	-	+	NON	Her2amp
LY2				+	-	-	NON	Luminal A

### Samples are classified as different subtypes

Breast cancer classification, in clinic, is measured according to these features: histological type, tumor grade, lymph node status and markers, such as oestrogen receptor (ER), progesterone receptor (PR) and human epidermal growth factor receptor 2 (HER2) [4, 6]. Breast cancer could be classified into at least four subtypes known as Luminal A, Luminal B, HER2-enriched and Basal-like (triple negative,TN), according to molecular characteristics which are summarized in Table 2.

PAM50 (Prediction Analysis for Microarrays) test is a risk model to identify the intrinsic subtypes in recent 5 years according to 50 gene expressions, including gene ESR1(ERα), PGR(PR) and ERBB2(HER2) [4]. This technique is based on Nano-string counter technology [38, 39]. PAM50 analysis was performed in R following the instructions therein [40]. Here, a threshold of 4.0 was chosen based on the false discovery rate, resulted in the 50-gene classifier. For the sake of missing data imputation, the status of ER, PR, HER2 and the PAM50 subtype calls were regarded as the subtype’s classification reference of breast cancer in this paper. If the sample status of ER, PR, and HER2 is known, samples classification of breast carcinoma is referenced to Table 2. Otherwise its subtype is assigned by mRNA gene expression-based PAM50 prediction, Additional file 1: Tables S1 and S2 provide all the classification information.

### Data processing

#### mRNA expression analysis and clustering between cell lines and tumors

All raw files of microarray mRNA expression, in the form of ‘CEL’ files, were downloaded from GEO GSE36133 and GSE41998. These raw data were normalized by the Affymetrix Microarray Suite 5.0 (MAS5.0) algorithm in accordance with background adjustments, scaling, and aggregation to remove non-biological elements of the signal. Common 22,267 probe sets, corresponding to 14,970 genes, are used comparison analysis for cell line and tumors. All samples in cell lines and tumors are divided into four subtypes group based on ER, PR, HER2 status: luminal A, luminal B, HER2-enrichment and Basal-like as the description before had shown in Additional file 1: Tables S1 and S2. Mean correlation value was obtained for each cell line and tumor in R platform by Pearson correlation analysis. Hierarchy clustering is analyzed between cell lines and tumors of breast cancer in GENE-E software.

#### DNA copy number data analysis

A total copy number of changes of TCGA 1033 tumors and CCLE 59 cell lines was detected using Affymetrix 6.0 single nucleotide polymorphism array (SNP 6.0 array) across 28,918 genes. Copy number was measured by a probe corresponding to a segment. They were then inferred and normalized based upon specific linear calibration curves. The circular binary segmentation (CBS) algorithm was used to normalize the segmentations (generally, log_2_(CN/2)) for further analysis. These segmentations were used to identify focal amplification/deletions and arm-level gains.

##### Fraction genome altered calculation

CNVs correspond to relatively large regions of the genome that have been deleted and inserted. To quantitate the extent of the genomic instability in each sample, we calculated the Fraction of Genome Altered (FGA, the fraction of genome lost and gained) as formula (1). The equation represents that sum lengths of all segments (*L*(*i*)) whose copy number (CN) segment is above the set threshold (*T*) and divide by sum of lengths of all segments (*L*(*i*)) [8]. Hence, the length of a segment having value equal to or greater than a set threshold are added and are divided by the sum of length of all segments.

1 $$ FGA={\displaystyle \sum_{\left|\mathrm{C}\mathrm{N}\mathrm{i}\right|>T}L(i)/{\displaystyle \sum L\left(\mathrm{i}\right)}} $$

Here, the threshold *T* is set to 0.2 for tumor samples and 0.3 for CCLE cell line samples. The threshold values are based on the average distribution density after samples CNV analysis. Cell lines always keep a copy number hyper-mutation degree than tumors’.

##### Copy number correlation calculation

With the help of Bioconductor package called ‘CNTools’ [41], these segments are mapped to corresponding gene region across 28,918 genes for both TCGA data and CCLE data, segments file is converted into gene files,then is used for next step correlation analysis. In order to reduce data contamination, only select the top 10 % CNV in 2094 genes segments mean for cross-Pearson’s-correlations calculation between 58 cell lines and 1049 tumors.

#### DNA exome mutation analysis

The mutation data was obtained directly from DNA sequence mutation annotation format (.maf) files where Illumina GA platform is used to test. In TCGA, 997 breast invasive cancer Level 2 somatic data is bulk downloaded and hybrid capture 1650 genes in CCLE 59 samples are obtained. According to software ANNOVAR gene-based annotation [21], gene mutation function is reported according to the 1000 Genomes Project and dbSNP database, somatic and germline mutation are identified in CCLE. Mutations are limited to somatic mutations and functional mutations. Hence intronic, silent and other mutations were ignored and only exonic mutations were considered.

##### Mutation frequency calculation

Gene mutational frequency can be described as a ratio of total number of gene mutations in samples to total number of samples. Actually, it is the measure of gene mutations probability in the breast cancer population.

##### Mutation rate calculation

The mutation number of bases for TCGA are detected from the bed files. The bed file contains a number of bases covered for each chromosome, in form of start and end location. Subtracting end from start gives number of bases covered by the reads. All bases obtained for each sample are summed together to obtain a whole number of bases covered, it is the given sample mutations rate per million bases (Mb). Bed files derive from ‘Wig’ format file. ‘Wig’ provides the number of reads for each region. In case of CCLE, the file can be downloaded from CCLE data portal. To TCGA, it is available from Synapse websites, a research-sharing platform (https://www.synapse.org/#!Synapse:syn1695394). Hence samples or gene mutations rates can be calculated through summing up all bases where read covered as mutations per Mb.

##### Mutation allele spectrum calculation

The patterns of six trans-version distributions were searched in the sequence annotation files from CCLE and TCGA irrespectively by R programming. Then, the mutation allele mode was obtained in each of the subtypes of breast tumors and cell lines. The correlation was calculated as mutation allele spectrum in each subtype between cell lines and tumors by Pearson-correlation method.

#### Proteins phosphorylation expression analysis and clustering

All basal phosphorylation and protein abundance data were obtained by RPPA technology from reference [19] and TCGA. There are 70 phosphoproteins across 38 cell lines of breast cancer that were generated by RPPA technology and pre-processed by the Gordon Mills lab at MD Anderson. Seventy phospho-proteins in 197 patient’s tumor of breast cancer were collected from TCGA in its Level 3 dataset. The common 50 protein expressions across 38 breast cancer cell lines and 197 TCGA tumors were used as comparison analysis between cell lines and tumors. The Pearson correlation method and hierarchy clustering was used to analyze and compare the similarity and non-similarity between cell lines and tumors in breast cancer. The result about how cell lines are close to its corresponding tumors are shown in Additional file 1: Table S6 based on 50 phosphor-proteins. In mRNA and its 50-protein phosphorylation comparison for cell lines and tumors, a gene has multiple isoforms while a protein phosphorylation has multi-sites. All forms of mRNA and its phosphorylation protein are compared with Pearson correlation, 38 genes’ average correlation coefficient was calculated and compared between cell lines and tumors in Fig. 11.

#### The cell line suitability score with breast tumors

The extent to which the breast cancer cell lines match genetic characteristics shared by the TCGA tumors was assessed using a whole *score* by formula (2). The score can catch a cell line’s whole similarity by four molecular profiles feature to tumors in breast cancer.

2 $$ Score=\mathrm{A}+\mathrm{B}+\mathrm{C}+\mathrm{D} $$

Where A is the gene expression similarity between cell lines and tumors by Pearson-correlation; B is the correlation with CNV segment mean of breast tumors; C is the correlation of genes mutation variation with breast tumors; D is the protein expression-based correlation with tumors in breast cancer. The score serves to identify a better or poorer cell lines model of breast cancer in entity molecular level and rank the graduate.

### Software tools

All data arranging was operated on Ubuntu Linux operating system by shell scripting programming. R and MATLAB was used to perform statistical analysis and plotting graphs [42]. Integrative Genomics Viewer (IGV) tools help to visualize large integrated data sets in a single frame and also supports zooming in to a particular chromosome or a certain region of the chromosome, and thus IGV (version 2.3) was used to create copy number profile plots [43]. GENE-E is a matrix visualization and analysis platform designed to support visual data exploration. Hierarchy clustering analysis used by GENE-E software on website www.broadinstitute.org/cancer/software/GENE-E/.

## Abbreviations

CCLE, cancer cell line encyclopedia; CNV, copy number variation; ER, estrogen receptor; FGA, fraction genome altered; GEO, gene expression omnibus; HER2, human epidermal growth factor receptor 2; IGV, integrative genomics viewer; MAS5, affymetrix microarray suite 5.0; NCCN, National Comprehensive Cancer Network; PAM50, prediction analysis for microarrays 50; PR, progesterone receptor; RPPA, reverse phase protein array; SNP, single nucleotide polymorphism array

## Additional files

Additional file 1: Table S1. The list of TCGA tumor samples used on each platform with associated subtype calls from each technology platforms, and clinical data. Table S2. The list of cell lines samples used on each platform with associated subtype calls from each technology platforms, and its annotation data. Table S3. The list of tumors samples from GEO used on gene expression comparison with associated ER, PR, HER2 status. Table S4. Mutation rate per Mb in cell lines and tumors in breast cancer. (Common genes). Table S5. Top 10 % genes of copy number variation in cell lines and tumors. Table S6. The comparison of phosphorylation protein vs gene expression in cell lines and tumors. (XLSX 2941 kb) Additional file 2: Table S7. Correlation coefficient *r* across 4 genomics level comparison in breast cancer. (XLSX 1527 kb)
